# A 3D Coverage Algorithm Based on Complex Surfaces for UAVs in Wireless Multimedia Sensor Networks

**DOI:** 10.3390/s19081902

**Published:** 2019-04-22

**Authors:** Jingyu Ru, Zixi Jia, Yufang Yang, Xiaosheng Yu, Chengdong Wu, Ming Xu

**Affiliations:** 1 School of Information Science and Engineering, Northeastern University, Wenhua Road, Heping District, Shenyang 110819, China; rujingyu@stumail.neu.edu.cn (J.R.); xuming@stumail.neu.edu.cn (M.X.); 2 School of Robot Science and Engineering, Northeastern University, Wenhua Road, Heping District, Shenyang 110819, China; jiazixi@ise.neu.edu.cn (Z.J.); yuxiaosheng@mail.neu.edu.cn (X.Y.); 3 Validation Center of Chery Jaguar Land Rover Company, Hongqiao Road, Changning Dstrict, Shanghai 201103, China; yufang.yang@cheryjaguarlandrover.com

**Keywords:** wireless multimedia sensor networks, sensor model, cuckoo search algorithm

## Abstract

Following the development of wireless multimedia sensor networks (WMSN), the coverage of the sensors in the network constitutes one of the key technologies that have a significant influence on the monitoring ability, quality of service, and network lifetime. The application environment of WMSN is always a complex surface, such as a hilly surface, that would likely cause monitoring shadowing problems. In this study, a new coverage-enhancing algorithm is presented to achieve an optimal coverage ratio of WMSN based on three-dimensional (3D) complex surfaces. By aiming at the complex surface, the use of a 3D sensing model, including a sensor monitoring model and a surface map calculation algorithm, is proposed to calculate the WMSN coverage information in an accurate manner. The coverage base map allowed the efficient estimation of the degree of monitoring occlusion efficiently and improved the system’s accuracy. To meet the requests of complex 3D surface monitoring tasks for multiple sensors, we propose a modified cuckoo search algorithm that considers the features of the WMSN coverage problem and combines the survival of the fittest, dynamic discovery probability, and the self-adaptation strategy of rotation. The evaluation outcomes demonstrate that the proposed algorithm can describe the 3D covering field but also improve both the coverage quality and efficiency of the WMSN on a complex surface.

## 1. Introduction

With the development of embedded systems and communication technology, wireless multimedia sensor networks (WMSN) have assumed a prominent scientific position [1,2]. Accordingly, WMSN constitute a winning solution with their information acquisition method and processing technology, and allow the acquisition of complete information in the monitoring area, especially video data [3]. They have been extensively used in different applications, such as military affairs, public security, environmental monitoring/forecasting, home automation, and in intelligent transportation [4,5,6].

Owing to the importance of WMSN, researchers have conducted extensive studies in this area [7,8]. Most of the existing studies have concentrated on localization [9], barrier coverage [3], data fusion [10], and sensor deployment [11]. Among these studies, the sensor network deployment problem guarantees the coverage of the monitoring area corresponding to the sensors, and provides the foundation of all the upper-layer applications.

The sensor network deployment problem is addressed by setting the position and direction of the sensors to monitor a region-of-interest. In former studies, most of the coverage studies had been based on the omnidirectional sensing model. Using this model, the sensors in WMSN can monitor the information in the perimeter of a circle with the sensor located at its center. Moreover, in view of the increasing number of requests for the applied condition and the variety of sensing information, the direction-oriented sensing model has drawn increased attention. It is assumed that the sensor could focus on a single primary direction at specific angles [12]. Some of the researchers based their studies on three-dimensional (3D) spherical sensing models which could be used underwater. Some studies have focused on the coverage effects on the ground. Accordingly, owing to the physical truth of the virtual sensors, such as the camera or video monitor, a specific type of a 3D directional sensing model was proposed [13]. The studies mainly focused on methods which were used to change the position or angle to maintain the ground coverage. The detection spaces in most of these studies were two-dimensional (2D), and simplified the network tasks owing to the decreased dimensionality. Some of the studies aimed at solving the problem in 3D space by considering the monitoring area as a planar surface. However, coverage studies for complex surfaces based on 3D sensing models have not been investigated.

It is highly likely that the understanding of the impact of a complex surface on WMSN will improve the monitoring validity and efficiency. For example, when the monitored area of WMSN is a mountainous region, a complex surface coverage model could represent adequately the complex surface information which can be used to improve the monitoring coverage rate. Furthermore, the knowledge of the obstructed visual field of the monitoring sensor caused by the complex surface can be utilized to help adjust the directions of the sensors, and can thus promote the efficiency of the network coverage.

Analyzing the WMSN’s deployment on a complex surface is challenging owing to the following three reasons. First, the problem of efficient integration of the coverage area of the sensor that monitors a complex surface is not trivial. Second, the complex surface may obstruct the visual field of a monitor that cannot be ignored; otherwise, it would significantly impact the precision of the coverage system. Third, the problem of setting multiple sensor orientations to obtain an optimal coverage ratio needs to be solved.

To address and resolve these challenges, we propose a novel framework for a complex surface coverage model for WMSN which (i) calculates the complex surface coverage and (ii) adjusts the directions of the sensors based on monitoring. Specifically, the contributions of our work are as follows:

- A 3D complex surface sensing model is proposed that reflects the real sensing area of the camera sensors. The model extracts the complex surface-related information based on a set of rules that satisfy the precision demands and efficiency
- Considering the fact that the monitoring area may be obstructed by physical barriers, such as hills or buildings, we propose a coverage-based map to help solve this problem. This model provides an achievable shade rule which quantifies the actual sensing area for each sensor.
- We propose a modified cuckoo search algorithm (MCSA) to compute the coverage area of the complex surface based on the survival of the fittest, dynamic discovery probability, and the self-adaptation strategy of rotation. Based on different parameter settings, our sensing models can be used for practical applications. The probabilistic algorithm is also adequate for the deterministic and stochastic deployment of WMSN.

The rest of the study is organized as follows: Section 2 introduces the motivation. In Section 3, the model design definitions of the 3D sensing model, coverage base map and determination of coverage are presented. In Section 4, a modified cuckoo search algorithm for the complex surface is proposed to solve the WMSN optimization problem. The simulation experiments and evaluation are outlined in Section 5. Related studies are presented in Section 6. Finally, the conclusions and prospects for future work are presented in Section 7.

## 2. Motivation

### 2.1. Benefits of Sensing Deployment

Compared to traditional sensors, camera sensors can provide more information of the environment in the forms of images or videos, and thus possess tremendous potential for use in various scenarios. For example, cameras which are fixed on the pole or on unmanned aerial vehicles (UAVs) can monitor hot-spot regions, detect intruders, and perform hazard assessments. The optimization of the spatial coverage is a key for mission requirements for monitoring tasks. In this study, the requirement is to identify the optimal orientation of the sensors in the region-of-interest [14]. The deployment of sensors with superior quality can prolong the network’s operating time and thus collect additional information.

### 2.2. 3D Coverage Algorithm on Complex Surfaces

Even though the sensing deployment has many benefits, there are still some difficulties in the efficient implementation of the system.

**Figure 1:**
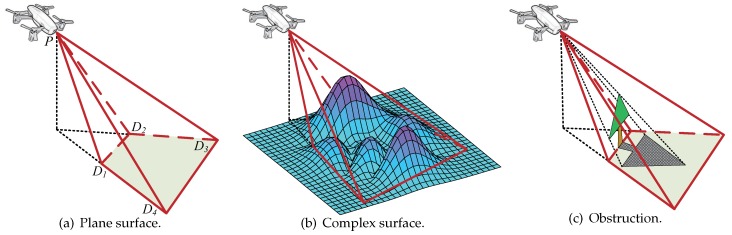
Schematics exemplifying the need for the use of 3D coverage models on complex surfaces.

First, most of the sensing models in this coverage study are based on planar surfaces and are modeled as sector-sensing or trapezoid models [15]. While the monitoring area is a plane, the projection is a trapezoid $D_{1}D_{2}D_{3}D_{4}$, as shown in Figure 1a. However, considering that in real tasks, the environment could normally be a mountainous surface or a small hill, the deployed areas of the WMSN is a 3D space that covers a nonplanar surface, as shown in Figure 1b. Considering the heavy computational burden associated with the calculation of the monitoring area [16], we need to define an approximate method to calculate the curved surface area that could take consideration of precision and efficiency.

Second, obstructions, such as trees or braces, may generate blind spots as shown in Figure 1c. Rapid and accurate calculation of the visual blind area is necessary to the stabilization of the WMSN. What is more important is the relative position of the sensor, and the fact that the obstruction will also influence the area of the blind spot that will impact the coverage strategy of the network.

Third, the overlap of the monitoring area of multiple multimedia sensors will cause system resource redundancy and a low-monitoring efficiency. To obtain a superior coverage ratio, we need to optimize the directions of the sensors.

We can now see that in the real environment, the complex surface will affect the effectiveness of the WMSN coverage. Thus, we need a 3D coverage algorithm based on a complex surface to optimize the deployment of the WMSN to improve the system’s efficiency.

### 2.3. Challenges

There are many challenges associated with the implementation of the system mentioned above. All of these challenges may lead to an inefficient system.

- The 3D sensing model for the calculation of the coverage area is complex. On the basis of different backgrounds, we need to analyze the sensing model from different directions. Building a 3D sensing model for every sensor is not an easy task. Even though the calculation of the sensing model and surface function of different terrains will produce accurate results, there is still a significant computational problem because the directions of the sensors are not fixed
- Any obstruction on the complex surface will reduce the monitoring efficiency. In a real WMSN system, the relations among sensor, obstruction, and raw complex surface data, are always intricate and expensive to manipulate and analyze. Moreover, obstructions impose different impacts because of the different heights and distances to the sensor
- Optimizing the directions of multiple sensors is not an easy task. Rotating any of the sensors may overlap the coverage areas from other sensors or may miss the monitoring of key areas, thereby influencing the monitoring outcome

Therefore, the analysis of the coverage of multiple sensors on complex surfaces by considering the existence of obstructions is difficult.

## 3. Model Design

### 3.1. 3D Sensing Model

The 3D sensing model consists of the sensor monitoring model that calculates the monitoring area of the sensor, and the surface map calculation that is used to calculate the area of the complex surface.

#### 3.1.1. Sensor Monitoring Model

**Figure 2:**
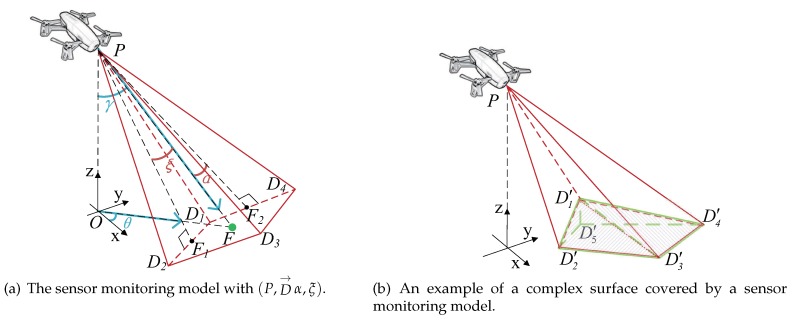
Sensor Monitoring Model.

The sensor monitoring model is denoted by four-tuple $\left(P,\;\vec{R}\;\;\alpha ,\;\xi \right)$ as Figure 2 shows. *P* denotes the coordinates of the sensor’s position in 3D space. Assume that the spatial surface that the monitor can effectively detect is a rectangular pyramid $P-D_{1}D_{2}D_{3}D_{4}$, which has a limit detection surface of $D_{1}D_{2}D_{3}D_{4}$ with *F* being the center point. Additionally, $\vec{R}\;=\left(\gamma ,\;\theta \right)$ denotes the main sensing direction $\bar{PF}$, where γ denotes the angle between $\bar{PF}$ and the negative direction of the Z–axis. In order to get better coverage results, the cameras adapt their angles to maximize the area on the surface. In this paper, we assume that different capture angle will introduce the same image resolution. The length of PF is referring to the detection range of the sensor, where θ is the angle of the anticlockwise rotation from the positive direction of the X–axis to OF. OF is on the horizontal surface of *F*, and OF is the shortest distance from *F* to the *Z*–axis. $F_{1}$ is the middle point of $D_{1}D_{2}$, and $F_{2}$ is the middle point of $D_{1}D_{4}$. Accordingly, $\xi =∠F_{1}PF$ and $\alpha =∠F_{2}PF$. This means that the sensor can monitor the area in the distance range of [θ−α,θ+α] and [γ−ξ,γ+ξ]. From this figure, we can calculate that

(1) $$PF_{1}=PF/\cos\xi$$

(2) $$D_{1}D_{2}=D_{3}D_{4}=2PF\cdot \tan\alpha$$

(3) $$D_{2}D_{3}=D_{1}D_{4}=2PF\cdot \tan\xi$$

The surface that the sensing model covers is defined as a complex surface. The monitoring area can be expressed as a function z=f(x,y) in the Cartesian coordinate system. When f(x,y)≠c, where *c* is a constant, the surface is a nonplanar surface. For example, Figure 2b depicts an example of monitoring a complex surface. In the figure, $D_{1}^{\prime }-D_{2}^{\prime }D_{3}^{\prime }D_{4}^{\prime }D_{5}^{\prime }$ is a rectangular pyramid. The area $D_{1}^{\prime }D_{2}^{\prime }D_{3}^{\prime }D_{4}^{\prime }$ is the monitored region-of-interest of the sensor *P* and is a complex surface.

We suppose a particular scene for our work where the camera sensor needed to capture the intruder’s face in the coverage area. To get a clear picture of the intruder’s face that could meet the needs of face recognition, we assume that the γ should be a non-zero angle because a proper inclination of the camera can improve the accuracy of the face recognition.

#### 3.1.2. Surface-map Calculation

We use a surface map to calculate the area of the complex surface. We first mesh the surface and calculate the area of each grid.

Each complex surface is meshed by a base grid surface *G* that consists of n×m square sets $G_{1,\;1},\;G_{1,\;2}\ldots G_{n,\;m}$. The *G* consists of (n+1)×(m+1) vertices that are defined in the form of the point set $L_{1,\;1},\;L_{1,\;2}\ldots \;L_{n+1,\;m+1}$, which constitute the intersection points of the grids.

**Figure 3:**
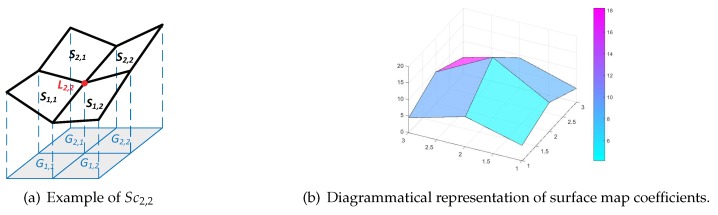
Surface map calculation.

Each point $L_{i,\;j}$ can be considered as an average area center point of the quadrangles around it. To link the four quadrangles to the $L_{i,\;j}$, we define a surface-map coefficient $Sc_{i,\;j}$ as follows.

(4) $$Sc_{i,\;j}=\frac{S_{i,\;j}+S_{i-1,\;j}+S_{i,\;j-1}+S_{i-1,\;j-1}}{4}$$

where $S_{i,j}$ is the area of the complex surface according to grid $G_{i,\;j}$. Among them, the $S_{0,\;j}=S_{i,\;0}=S_{n+1,\;j}=S_{i,\;m+1}=0$. For example, in Figure 3a, the value of $L_{2,\;2}$ is surrounded by the area of $S_{1,\;1},S_{1,\;2},S_{2,\;1},S_{2,\;2}$.

Each complex surface has a specific and fixed surface map. For example, as Figure 3b shows, it is the diagram of a surface map coefficient matrix of the complex surface in Figure 3a. The higher the value of $Sc_{i,j}$ is, the more area it represents, and the steeper the surrounding areas are.

### 3.2. Coverage Base Map

**Figure 4:**
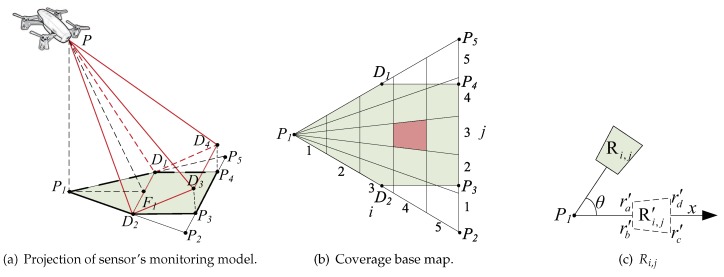
Coverage base map construction.

We build a coverage base map to help estimate the impact of the shadowing by the complex coverage of each sensor. The coverage base map is built based on the projection of the sensor’s monitoring model, as shown in Figure 4a. In this figure, considering that the sensor monitoring model is always a symmetrical pyramid with a rectangular base, its projection on the XOY is restricted to the triangle $▵P_{1}P_{2}P_{5}$ that is determined by the edge of the sensor’s monitoring model. In addition, the practical coverage of the sensor’s monitoring model consists of an isosceles triangle $▵P_{1}D_{1}D_{2}$ and a rectangle $D_{1}D_{2}P_{3}P_{4}$, denoted as the green polygon $P_{1}D_{2}P_{3}P_{4}D_{1}$.

(5) $$D_{2}P_{3}=D_{1}P_{4}=D_{2}D_{3}\cdot \cos\gamma =2PF\cdot \tan\xi \cos\gamma$$

According to Equation (1), we have

(6) $$PP_{1}=PF_{1}\cdot \cos\left(\gamma -\xi \right)=PF\cdot \cos\left(\gamma -\xi \right)/\cos\xi$$

(7) $$P_{1}F_{1}=PF_{1}\cdot \sin\left(\gamma -\xi \right)=PF\cdot \sin\left(\gamma -\xi \right)/\cos\xi$$

where $P_{1}F_{1}$ is the height of the $▵P_{1}D_{1}D_{2}$. Considering Equation (2),

(8) $$∠P_{2}P_{1}F_{1}=\arctan\frac{D_{1}D_{2}/2}{P_{1}F_{1}}=\arctan\left[\tan\alpha \cos\xi /\sin\left(\gamma -\xi \right)\right]$$

Accordingly, we have that

(9) $$\begin{array}{cc} P_{1}P_{2}=P_{1}P_{5} & =\left(P_{1}F_{1}+D_{2}P_{3}\right)/\cos\left(∠P_{2}P_{1}F_{1}\right)\\ & =PF\frac{2\sin\xi \cos\gamma \sin(\gamma -\xi )+1}{\cos\xi \sin\left(\gamma -\xi \right)\cos\left(∠P_{2}P_{1}F_{1}\right)} \end{array}$$

(10) $$P_{2}P_{5}=2\sin∠P_{2}P_{1}F_{1}\cdot P_{1}P_{2}$$

For the sake of simplicity and improved understanding, parts of the coordinate calculation are placed into the Appendix. We use a point-in-triangle-test algorithm in [17] to select all the points $L\in ▵P_{1}P_{2}P_{5}$ as the alternative base map of the sensor’s monitoring model, as shown in Figure 4b. We then divide this alternative base map equally to build a 5×5 gridding configuration in the XOY plane. Firstly, we split the $▵P_{1}P_{2}P_{5}$ into five parts by dividing $P_{2}P_{5}$ into five equal segments, and we connect the vertex $P_{1}$ to both ends of the segments. Thus, we divide each part into five subregions by four parallel equidistant straight lines. This means that the width of the subregion is $R_{w}=\left(P_{1}F_{1}+D_{2}P_{3}\right)/5$. For example, in Figure 4b, the red trapezoid is represented by $R_{4,\;3}$.

Considering that the real monitoring range is defined by the polygon $P_{1}D_{2}P_{3}P_{4}D_{1}$, as shown in Figure 4b, the *L* in $▵D_{2}P_{2}P_{3}$ and $▵D_{1}P_{4}P_{5}$ should be removed. Finally, the average *z* of the *L* in each $R_{i,\;j}$ is defined as $H_{i,\;j}$ that is used to denote the height of the complex surface in $R_{i,\;j}$. Accordingly, the summation of Sc of *L* for each $R_{i,\;j}$ is defined as $Sr_{i,\;j}$ that is used to represent the area of the complex surface in $R_{i,\;j}$.

As shown in Figure 4c, the $R_{i,j}$ is rotated θ anticlockwise from the $R_{i,\;j}^{\prime }$ on the X–axis with $\theta =0^{\circ }$. The coordinates of the four vertices $x_{r_{a}},\;x_{r_{b}},\;x_{r_{c}},\;x_{r_{d}}$ of $R_{i,\;j}$ and $x_{r_{a}^{\prime }},\;x_{r_{b}^{\prime }},\;x_{r_{c}^{\prime }},\;x_{r_{d}^{\prime }}$ of $R_{i,j}$ are calculated in the Appendix A.

### 3.3. Determination of Coverage

In this part we describe the distinction of coverage on the complex surface. The distinction of coverage is the coverage criterion based on the 3D sensing model and the coverage base map.

The sensor *P* in Figure 4a is the sensor with coordinates $\left(x_{p},\;y_{p},\;z_{p}\right)$. $P^{\prime }$ is the projection of *P* on the curved surface with coordinates $\left(x_{p},\;y_{p},\;z_{p^{\prime }}\right)$ where $z_{p^{\prime }}=z_{p}-h$ and $PP^{\prime }=h$ is the height of the sensor. *L* is an arbitrary point on the complex surface with $\left(x_{l},\;y_{l},\;z_{l}\right)$, where $z_{l}$ is the $H_{i,\;j}$ of $R_{i,\;j}$ that *L* belongs to. $L^{\prime }$ is the projection of *L* on the plane of $P^{\prime }$ with $\left(x_{l^{\prime }},\;y_{l^{\prime }},\;z_{l^{\prime }}\right)$ and $z_{l^{\prime }}=z_{p^{\prime }}$. The $∠L^{\prime }P^{\prime }x$ is the angle determined by the anticlockwise rotation from the positive direction of the X–axis to $P^{\prime }L^{\prime }$. The values of ∠LPF, $∠L^{\prime }P^{\prime }x$ and $∠P^{\prime }PL$ can be calculated by the cosine formula.

There are two steps used to distinguish whether the point *L* is under the coverage of sensor *P*. The first step is used to distinguish whether *L* is in the coverage of the sensor monitoring model $P-D_{1}D_{2}D_{3}D_{4}$, and the second step is used to distinguish whether the obstruction blocks *L*.

• Determining the sensor monitoring model 

If the point *L* meets all the criteria listed below, we infer that *L* is covered by the sensor’s monitoring model for sensor *P*.

(i) PL·cos(∠FPL)<PF;

(ii) $|∠P^{\prime }PL-\gamma |<\xi$;

(iii) $|∠L^{\prime }P^{\prime }x-\theta |<\alpha$.

• Determining the obstruction 

**Figure 5:**
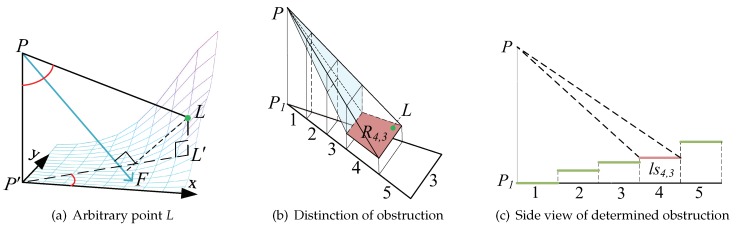
Determination of coverage.

Firstly, we identify the $R_{i,\;j}$ in which the point *L* belongs to, and we distinguish whether there exists an obstruction in the covered space from *P* to $R_{i,\;j}$, as shown in the blue highlighted area in Figure 5b. Considering the characteristic structure of the coverage base map in this study, we simplify the 3D sensor arrangement into a 2D problem by analyzing the side view of the sensor’s monitoring model, as shown in Figure 5c. Each $R_{i,\;j}$ is considered as a line segment $ls_{i,\;j}$ in the side view with a length equal to $R_{w}$. For each $R_{i,\;j}$ we determine whether they have intersections with the lines from *P* to both ends of $ls_{i,\;j}$ from $ls_{1,\;j}$ to $ls_{i-1,\;j}$ using the 2D specialization of the 3D line intersection algorithm [18]. If there is no intersection, we conclude that there is no obstruction which blocks the sensor for the monitoring of the *L*.

Given a sensor P(i), if the point *L* is covered by sensor P(i) and there is no obstruction, we define it as ζ(L)=1, else ζ(L)=0. For example, the *L* that shows as a green point in Figure 5a according to $R_{4,\;3}$ is represented by the red trapezoid in Figure 5b. Its side view is $ls_{4,\;3}$ and is shown in Figure 5b as a red-line segment. Additionally, the point *L* is covered by sensor *P* and there is no obstruction, thus ζ(L)=1.

Given that a complex surface consists of (n+1)×(m+1)$L_{i,\;j}$, the coverage ratio is defined as a real value η expressed as

(11) $$\eta =\frac{\sum_{i=1}^{n+1}\sum_{j=1}^{m+1}\zeta \left(L_{i,j}\right)\times Sc\left(L_{i,j}\right)}{\sum_{i=1}^{n+1}\sum_{j=1}^{m+1}Sc\left(L_{i,j}\right)}$$

## 4. Modified Cuckoo Search Algorithm

The cuckoo search (CS) algorithm is a bio-inspired algorithm which was proposed by Yang [19]. The CS algorithm is used for solving the optimization problem based on the way cuckoos lay their eggs [20,21,22,23,24].

### 4.1. Cuckoo Search for WMSN

Considering the character of the WMSN, the survival of the fittest, dynamic discovery probability, and the self-adaptation strategy of rotation are proposed to ensure that the modified CS algorithm (MSCA) satisfies the demands of the WMSN coverage problem. The basic steps of the MSCA can be summarized in Algorithm 1. The overall MSCA can be summarized by the following rules: (1) Each cuckoo lays one egg at the a time. (2) The best egg will be saved as the solution. (3) The egg will be randomly discovered by the host based on the dynamic discover probability $P_{a}\left(P\left(i\right),\;k\right)$. (4) The discovered egg will modify itself based on the self-adaptation strategy of rotation.

**Algorithm 1:** MSCA

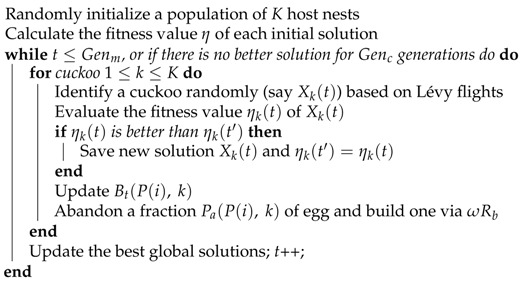



To present our work more effectively, we fixed the sensors at the height of *h* meters above the complex surface, and we kept the position and the values of γ of the sensors constant in the optimization process. The sensor nodes are randomly deployed and will not change their locations.

$X_{k}=\left[\theta_{1},\theta_{2},\theta_{3},\ldots \theta_{N}\right]$ reflects the θ of the *k*th nest with *N* sensors, while the number of nests is *K*, and the searching space for each sensor is [0,2π]. In the following parts, we optimize the value of θ to obtain the best coverage effectiveness η that is the fitness evaluation function $\eta_{k}\left(t\right)$.

In the MCSA, each egg in a nest indicates a solution *X*, and each cuckoo can only lay one egg. When a new solution $X_{k}\left(t+1\right)$ is generated for the *k*th cuckoo in the t+1th iteration, the following Lévy flight is performed as follows.

(12) $$X_{k}\left(t+1\right)=X_{k}\left(t\right)+\epsilon ⊕l\overset{´}{e}vy\left(\lambda \right)$$

where ϵ>0 is the step size, k=1,2,…M, where *M* indicates the population of the host nests, and *t* represents the current number of iterations. The product ⊕ is the entry-wise multiplication. $l\overset{´}{e}vy\left(\lambda \right)$ is an Lévy distribution function and is converted into a probability density function as follows.

(13) $$l\overset{´}{e}vy∼\mu =t^{-\lambda }\left(1<\lambda \leq 3\right)$$

where λ is a parameter which is the mean or expectation of the occurrence of the event during a unit interval. The step length can be calculated by using the following equation.

(14) $$l\overset{´}{e}vy\left(\lambda \right)=\frac{\mu }{{\left|v\right|}^{1/\lambda }}$$

where μ and *v* are drawn from the normal distributions as follows.

(15) $$\mu ∼N\left(0,\sigma_{\mu }^{2}\right),\;v∼N\left(0,\sigma_{v}^{2}\right)$$

where

(16) $$\sigma_{\mu }=\frac{\Gamma (1+\lambda \cdot \sin(\pi \lambda /2))}{\Gamma \left(\left(1+\lambda \right)/2\right)\cdot \lambda 2^{(\lambda -1)/2}},\;\sigma_{v}=1$$

Γ denotes the gamma function. The Lévy flight forms a random walk process with a power-law Lévy distribution with a heavy tail.

### 4.2. Survival of the Fittest

In our WMSN coverage problem, we only considered a single degree of coverage. Thus, the coverage overlap attributed to the multiple sensors should be avoided. Moreover, we considered the priority of preservation for the sensor that had a better coverage area.

Consider the sensors P(i) and P(j) with directions $\theta_{i}$ and $\theta_{j}$ at the *t*th iteration. The coverage of P(i) and P(j) is represented by $S_{t}\left(P\left(i\right),\;\theta_{i}\right)$ and $S_{t}\left(P\left(j\right),\;\theta_{j}\right)$, respectively. Their coverage overlap regions are defined as $O_{t}\left(P(i),\;P(j)\right)$. If $S_{t}\left(P\left(i\right),\theta_{i}\right)\leq S_{t}\left(P\left(j\right),\;\theta_{j}\right)$, we consider that the coverage of P(j) is fitter than those of the P(i)’s. $S_{t}^{\prime }\left(P\left(i\right),\;\theta_{i}\right)$ is defined as the survival coverage of $S_{t}\left(P\left(i\right),\;\theta_{i}\right)$ that is calculated by

(17) $$S_{t}^{\prime }\left(P\left(i\right),\;\theta_{i}\right)=S_{t}\left(P\left(i\right),\;\theta_{i}\right)-O_{t}\left(P(i),\;P(j)\right)$$

**Figure 6:**
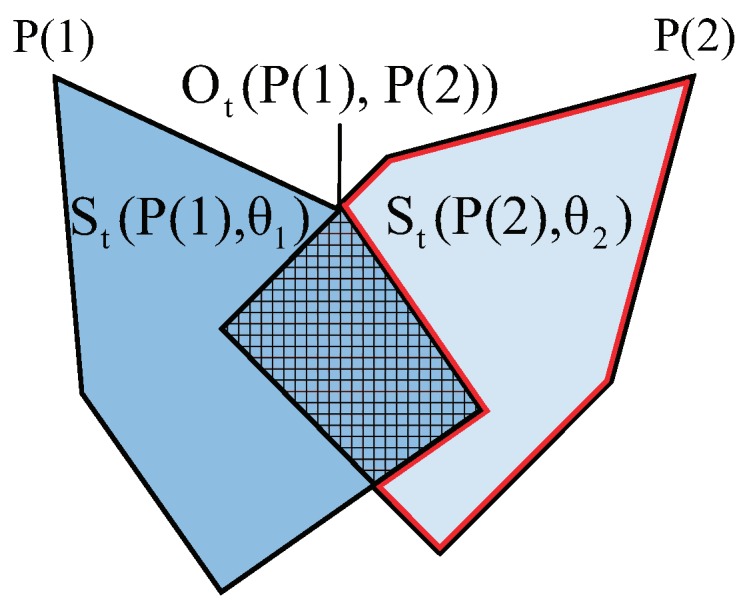
Survival of the fittest.

For example, in Figure 6 the projection of coverage of P(1) is represented by the dark blue polygon, and its area is $S_{t}\left(P\left(1\right),\;\theta_{1}\right)$. The coverage of P(2) is represented by the light blue polygon and its area is $S_{t}\left(P\left(2\right),\;\theta_{2}\right)$. Their coverage overlap regions are denoted by the shadow area, which is defined as $O_{t}\left(P(1),\;P(2)\right)$. Consider that $S_{t}\left(P\left(1\right),\;\theta_{1}\right)>S_{t}\left(P\left(2\right),\theta_{2}\right)$, $S_{t}^{\prime }\left(P\left(1\right),\;\theta_{1}\right)=S_{t}\left(P\left(1\right),\;\theta_{1}\right)$ does not change and $S_{t}^{\prime }\left(P\left(2\right),\;\theta_{2}\right)=S_{t}\left(P\left(2\right),\;\theta_{2}\right)-O_{t}\left(P\left(1\right),\;P\left(2\right)\right)$ is represented by the red polygon.

### 4.3. Dynamic Discovery Probability

In MSCA, a host can discover an alien egg based on the discovery probability $P_{a}$. Using $P_{a}$, the host bird can either throw the egg away, or abandon the nest $X_{k}$ to build a completely new nest in a new location.

The solution in each nest is multidimensional. We change the partial dimensions of the solution instead of giving up all of them. In other words, each sensor P(i) in the *k*th nest has its own independent dynamic discovery probability. From the perspective of coverage of WMSN, the sensor’s location and environment are unchanged. Thus, the coverage area of each sensor is not infinite. Ideally, the sensor with a higher $S_{t}^{\prime }\left(P(i),\;w_{i}\right)$ indicates that the $P_{i}$ with $w_{i}$ has a higher probability to be one of the components of the global optimum solution. We define $B_{t}\left(P(i),\;k\right)$ as the highest $S_{t}^{\prime }\left(P(i),\;\theta_{i}\right)$ value from the 1st to the *t*th iteration for the *k*th cuckoo. We define the dynamic discovery probability $P_{a}\left(P\left(i\right),\;k\right)$ as follows.

(18) $$P_{a}\left(P\left(i\right),\;k\right)=P_{b}\cdot S_{t}^{\prime }\left(P(i),\;\theta_{j}\right)/B_{t}\left(P(i),\;k\right)$$

where $P_{b}$ is the basic discovery probability.

The dynamic discovery probability makes it possible to (a) identify within a short time the part of the egg that is potentially not appropriate and (b) identify the optimum solution in a wild range.

### 4.4. Self-adaptation Rotation Strategy

After the random selection of the inappropriate part of the egg, we use sensor coverage information to guide the rotational direction.

**Figure 7:**
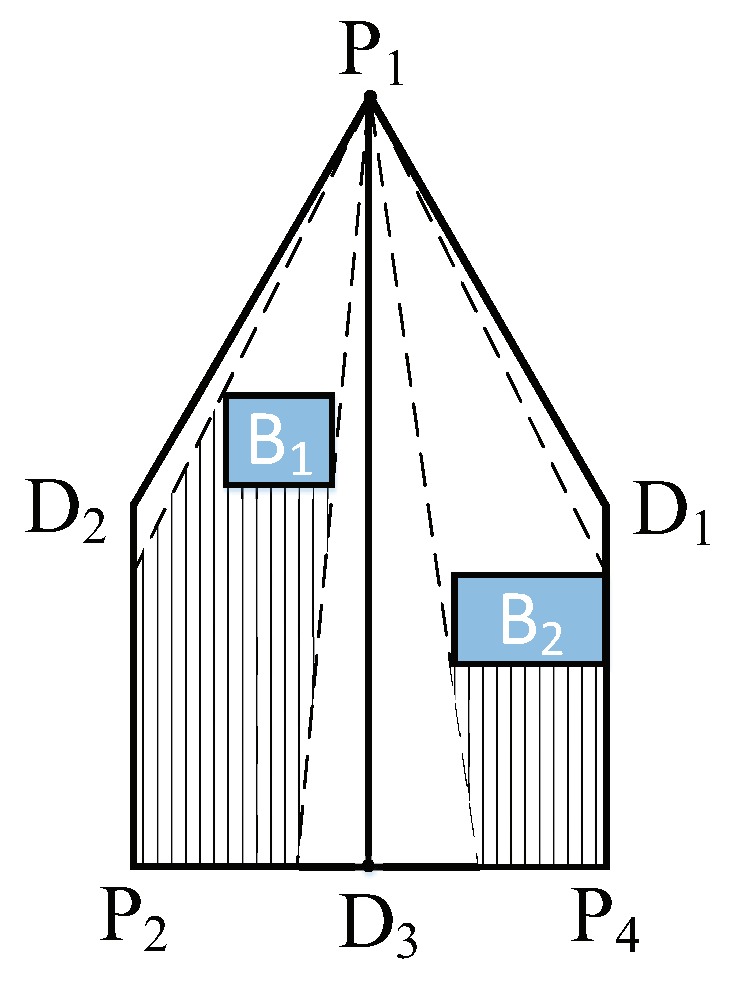
Self-adaptation rotation strategy.

Given the selection of a sensor P(i) for random rotation, the projection of its coverage is shown as the polygonal surface $P_{1}D_{2}P_{2}P_{4}D_{1}$ in Figure 7.

The rotational direction of the sensor is a binary selection either in the clockwise or anticlockwise directions. We define the clockwise direction as the direction of rotation in the positive direction to the sensor. Correspondingly, the anticlockwise direction is defined at the rotation in the negative direction to the sensor.

Accordingly, the projection of the sensor’s coverage $P_{1}D_{2}P_{2}P_{4}D_{1}$ is equally divided into a positive polygon $PP=P_{1}D_{2}P_{2}D_{3}$ and a negative polygon $NP=P_{1}D_{1}P_{4}D_{3}$. The $D_{3}$ is the intermediate point of $P_{2}P_{4}$. Both polygons may be blocked by the barriers. For example, in Figure 7, the $B_{1}$ and $B_{2}$ are barriers and the area they block is represented by the shadow in the figure. We define the coverage area in PP as Sc(PP) and the coverage area in NP as Sc(NP). This area can be calculated as follows:

(19) $$Sc\left(PP\right)=\sum Sc(L_{i,j}),\;\;L_{i,j}\in PP$$

(20) $$Sc\left(NP\right)=\sum Sc(L_{i,j}),\;\;L_{i,j}\in NP$$

From one viewpoint, we wish that the random rotation could reduce the potential blocking influences. Therefore, the sensor should randomly orientate itself along the direction with the fewer obstacles. From another viewpoint, we wish that the random rotation could cover a more complex surface.

Therefore, the barrier ratio $R_{b}$ is defined as follows

(21) $$R_{b}=\upsilon \cdot Sc(PP)/Sc(NP)$$

where υ is a referenced rotational direction that determines the angular velocity of rotation.

The $\theta_{i}^{\prime }$ is the new sensor direction that is calculated as follows.

(22) $$\theta_{i}^{\prime }=\left\{\begin{array}{c} \theta_{i}+\omega \cdot R_{b},\;\;\;\sum Sc(PP)\geq \sum Sc(NP)\\ \theta_{i}-\omega \cdot R_{b}^{-1},\;\sum Sc(PP)<\sum Sc(NP) \end{array}\right.$$

where ω∈[0,1] is a random number.

## 5. Evaluation

In this section, all the simulation experiments are carried out with MATLAB. The experiments were executed on a desktop with an Intel i7–6700 central processing unit (CPU) @ 3.40 GHz and with a 16 GB memory.

### 5.1. Surface Map

In this subsection, we evaluate the construction of the surface map.

**Figure 8:**
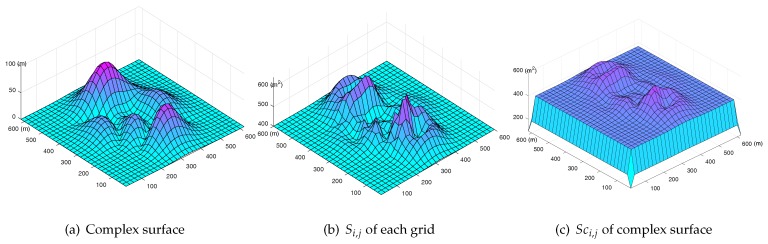
Construction of surface map.

The complex surface in this study is shown in Figure 8a. The monitoring area is 600 m × 600 m, is meshed by 30 × 30 grids, and has a length of 10 m. Figure 8b shows $S_{i,j}$ of the complex surface, which is approximately proportional to the gradient of the surface. Furthermore, the summit and the foot of the hill have lower $S_{i,j}$ values, while the side of the hill has a higher $S_{i,j}$ value. The coefficient of the surface-map is shown in Figure 8c. It is understandable that its fluctuation is lower than $S_{i,j}$ because $Sc_{i,j}$ is calculated based on the average value of $S_{i,j}$ around each $L_{i,j}$. The four sides of the surface maps have smaller $Sc_{i,j}$ values than the one in the middle because they only cover the area of the two grids around them. Similarly, the four corners of the surface map have the lowest $Sc_{i,j}$ values because they just cover the area of one grid.

### 5.2. 3D Coverage on Complex Surfaces

In this subsection, we evaluate the 3D coverage on the complex surface. Four sensors {P(1),P(2),P(3),P(4)} are installed on a UAV, and the XOY coordinates of the UAV are $P_{1}\left(220,\;460\right)$, $P_{2}\left(160,\;440\right)$, $P_{3}\left(160,\;80\right)$, and $P_{4}\left(580,\;200\right)$. According to Equation (8), the $∠P_{2}P_{1}F_{1}=125.3^{\circ }$. The parameters of the sensors are shown in Table 1.

**Figure 9:**
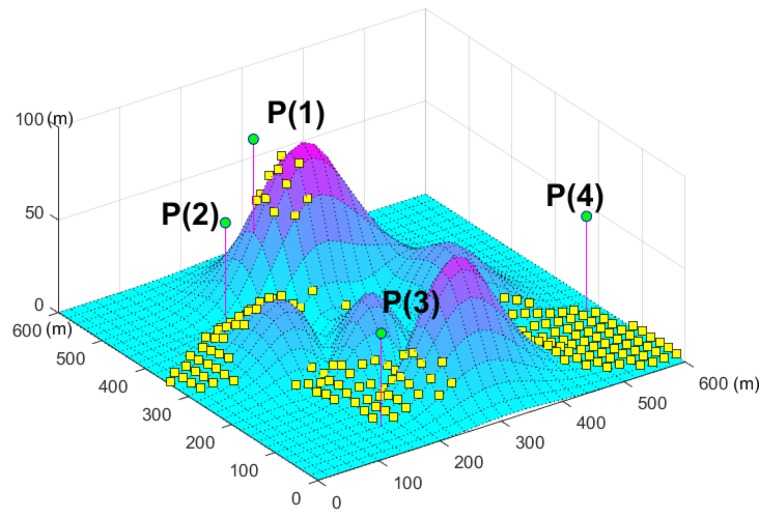
3D coverage example on complex surface.

The simulated result is shown in Figure 9.

In the figure, the green circles represent the sensors, the pink line is the height of the sensors, and the yellow squares represent the *L* that is monitored by the sensor.

The coverage areas of these four sensors have different characteristics. From this figure, we can conclude that the coverage area of P(1) is extremely small because the mountain peaks block most of this area. The coverage areas of P(2) and P(3) are similar on the hillside and the valley. The coverage area of P(4) is flat, and its shape is approximately triangular on the horizontal plane. This indicates that the 3D coverage model can estimate the different complex coverage areas in a precise and efficient manner.

### 5.3. Effectiveness of MSCA

In this subsection, we verify the effectiveness of MCSA using the 3D sensing model and the identification of coverage. All the parameter settings of the sensor and the environment are the same as the those reported in Section 5.2. The parameter settings of MSCA are shown in Table 2 as follows.

The sensors are placed in the area with random deployments and directions ω. Accordingly, we compared the MCSA with other algorithms, including the standard CS (SCS), particle swarm optimization (PSO), gene algorithm (GA), and MCSA without a coverage base map (MCSAnC). The SCS, PSO, and GA, are associated with coverage base maps.

**Figure 10:**
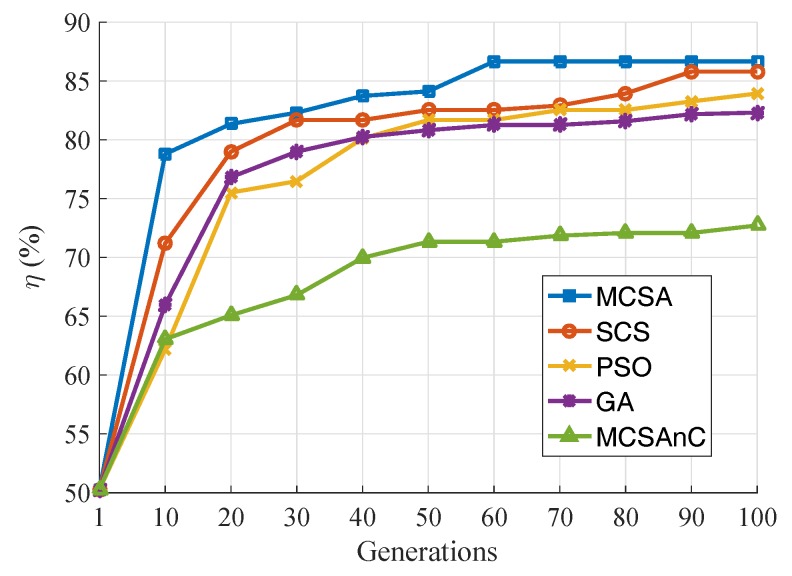
Plots of coverage-enhancing ratio as a function of generation number.

For a better comparison of the different algorithms, all the deployments, directions of the sensors, and the fitness functions for the five algorithms were chosen to be the same. The average initial coverage ratio of the tested five algorithms was η=50.3%. The number of populations of all the algorithms was 30. Figure 10 illustrates that the MCSA has the best coverage ratio equal to η=86.7% after 100 generations. In addition, SCS has a better η than PSO and GA. MCSAnC has the worst η because it ignores the existing barriers. Accordingly, it will never attain the value of the optimum coverage ratio. It can also be concluded that the MCSA has the best and optimum searching rate compared to the others. This illustrates that the MCSA contains the survival of the fittest and the dynamic discovery probability features. Additionally, the self-adaptation strategy of rotation can accelerate the local search process and help WMSN identify the best coverage ratio.

**Figure 11:**
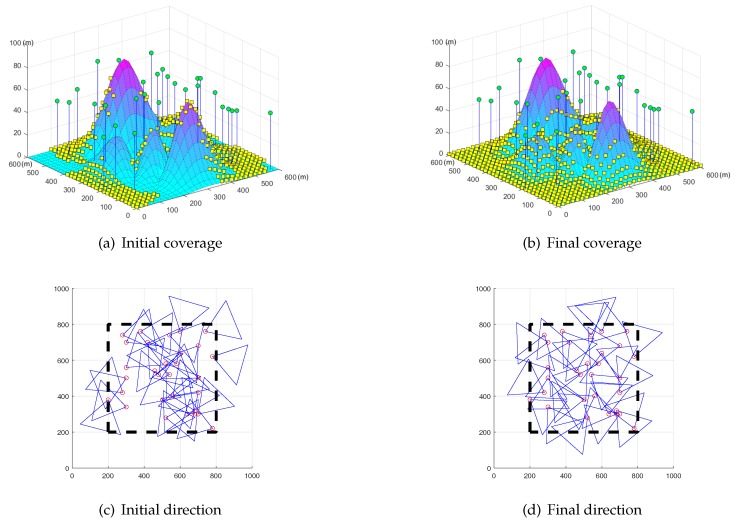
Example of MCSA on complex surface.

The example of the initial and the final coverage results of MCSA are shown in Figure 11a,b, respectively. Compared to the initial coverage, we can show that the MCSA can help the system which has the highest monitoring field.

Figure 11c,d are respective examples of the top views of the initial and the final directions of the sensors. The black dashed box is the monitored area. We can infer that the initial directions of the sensors contain a lot of overlap and some of them are oriented in directions outside the monitoring area. After the use of the MCSA, the coverage quality improves considerably such that the overlap of the coverage decreases and the orientations of the sensors become more reasonable.

We changed the number of sensors in the monitoring area from 10 to 20, 30, 40, and 50.

From the Table 3, we can infer that the initial and final η increase as the number of sensors increases, but the increment rate of the η decreases and N increases.

**Figure 12:**
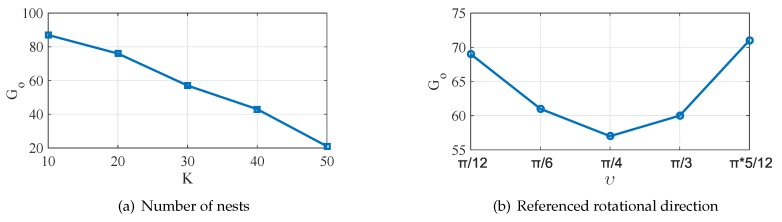
Comparison of variations of optimum generations as a function of the nest number and the referenced rotational direction.

We define $G_{o}$ as the generation at which WMSN identifies the optimum solution. All the other parameters do not change. We evaluate the relationship between the *K* and $G_{o}$ in Figure 12a. From the figure, we can observe that the WMSN will identify the best solution at a faster rate as the number of *K* increases.

The relationship between the referenced rotational direction υ and $G_{o}$ is shown in Figure 12b. When υ is approximately equal to π/4, the WMSN can identify the optimal solution in the fastest way. Otherwise, when υ increases or decreases, $G_{o}$ increases.

**Figure 13:**
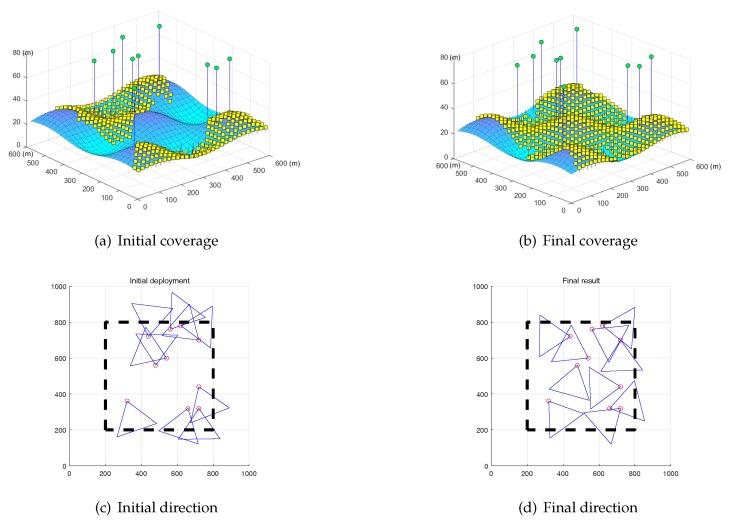
Example of MCSA on complex surface.

We evaluate the MCSA on a wave surface that is slightly flatter than the one before. The number of sensors is 10. In this situation, no obstruction could block the visual field of the monitoring sensor. The example of the initial and the final coverage results of MCSA are shown in Figure 13a with η=48.9% and Figure 13b with η=75.1%, respectively. Compared to the initial coverage η=48.9%, we can show that the MCSA can help the system which has the highest monitoring field.

Figure 13c,d are respective examples of the top views of the initial and the final directions of the sensors. We can infer that the MCSA can improve the coverage quality on either complex surface and flat surface.

**Figure 14:**
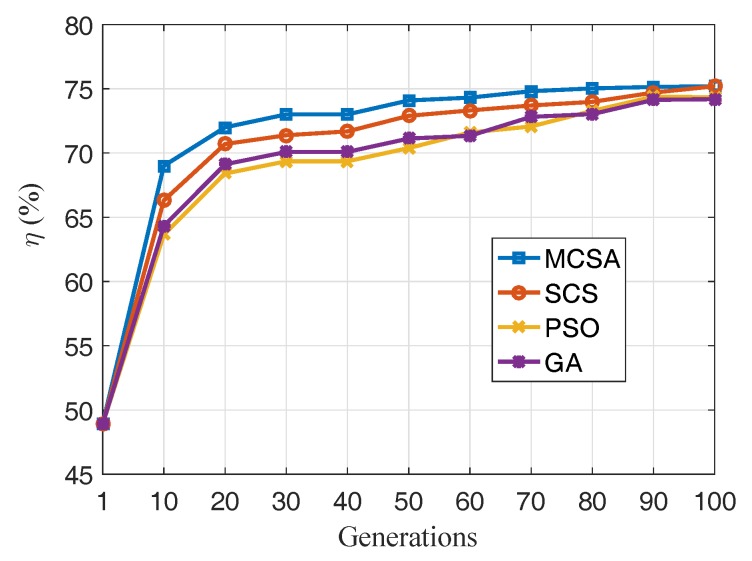
Plots of coverage-enhancing ratio as a function of generation number.

Figure 14 compare the coverage ratio of generation number with MCSA, SCS, PSO and GA. The figure illustrates that four algorithms have a similar best coverage ratio equal to η=75%. Also, it can be concluded that the MCSA has the optimum searching rate compared to the others. However, the advantage is not as obvious as the evaluations using a complex surface. It illustrates that the MCSA works as well as the others in the general surface, but it is more suitable for the complex one.

## 6. Related Studies

To effectively monitor the target area, it is necessary to summarize the sensor into the sensing model. Most of the coverage studies were based on the omnidirectional sensing model [25,26]. For example, in 2D space, the certainty coverage algorithm was based on grids, sleeping node protocols, etc. Zou [9] proposed an algorithm based on virtual force, the expression of the physical law is similar is similar to the gravitational and repulsive forces, and constitutes the topological structure of the entire network which is determined by the forces of each sensor. Bhuiyan [27] presented an approach referred to as fault tolerance in structural health monitoring, to guarantee a specific degree of fault tolerance. It searched the repaired points in clusters in a distributed manner and placed a set of backup sensors at these points in such a way that it satisfied the engineering requirements. Given the increasing number of requests for the applied condition and the variety of sensing information, the direction-oriented sensing model drew increased attention. Tao and Ma [28] proposed a distributed coverage-enhancing algorithm that aimed to reduce the overlapped area, and organized the subset network to decrease the energy output and reduce the computational complexity. Cheng [28,29] proved that the enhancement in a direction-oriented network was a complete NP problem, and proposed a distributed Greedy algorithm which selected a direction with the most prominent coverage in a limited area followed by the execution of local iterations to achieve the coverage goal. Heo and Wang [25] introduced potential virtual fields into a randomly deployed, coverage-enhancing problem, and applied it to both fully oriented and direction-oriented sensing models. Zhao [30] proposed an effective coverage enhancement method for surface coverage regarding the complex surface, but the study was still based on the traditional sensing model, and did not involve the 3D sensing model. Most of the prior studies were based on 2D space which ignored the heights of the sensors and lost the information within the real monitoring area. Ma [31] proposed a 3D sensing model and utilized the potential virtual field combined with a simulated annealing algorithm to define the sensing area. Cai [32] proposed a heuristic algorithm based on multiple directional cover sets. The algorithm identified multiple coverage sets without overlap but with full coverage to the target. This algorithm aimed to extend the lifetime of the network. As the number of sets increased, the network’s lifetime was prolonged. Huang [33] focused on 3D space under water. He proposed a control method that deployed the sensors evenly on the water surface and adjusted the depth using a distributed algorithm. Xiang [34] proposed the 3D space detection and coverage growing algorithm with the use of a probabilistic sensing model with five different collaborative detectors based on spatial correlation and signal detection theory. The aforementioned studies assumed that wireless sensor networks were applied in an ideal environment. However, 3D coverage should be effective in much more complex conditions during practical applications. To-this-date, there have been no published studies on 3D coverage enhancement algorithms in the presence of complex surface conditions.

## 7. Conclusions

In this study, we proposed a modified CS algorithm to optimize the coverage problem in WMSN using the 3D sensing model and the coverage base map. The 3D sensing model included the sensor monitoring model and the surface map calculation to rapidly build a 3D coverage model. The coverage base map was able to efficiently estimate the degree of monitoring occlusion and improved the system’s accuracy. In addition, based on the characteristics of WMSN, we modified the CS algorithm to include the survival of the fittest, dynamic discovery probability, and the self-adaptation strategy of rotation. Extensive experiments have been performed to study the availability of the 3D sensing model and the coverage base map. Additionally, a comparative study has been conducted, and experimental results proved that our MSCA performed better than other optimum algorithms, such as CS, PSO, and GA.

We know that some assumption such as the unmovable UAVs and unchangeable γ weaken the ability of the UAVs and the rationality for using UAV sensors, it is a limitation of our work and also a compromise our face-view scenario application, we hope to explore more usage scenarios in the future to expand the related research. In the future, we will focus on the design of an innovative system to test the MCSA in practical applications. Also, to find the best deployment strategy that has the fewest number of sensors and less limitation but the best coverage ratio is another topic that we will study in future work.

## Figures and Tables

**Table 1:** Sensor parameter settings.

Parameter Settings	Value
*h* of sensor	50 m
α of sensor	π/6
ξ of sensor	π/6
γ of sensor	π/4
Detection range PF	200 m

**Table 2:** Parameter settings of MCSA.

Parameter Settings	Value
Sensor number N	30
Number of nest K	20
Basic discovery possibility $P_{b}$	0.25
Referenced rotation direction υ	π/3
$Gen_{m}$	100
$Gen_{c}$	10

**Table 3:** Parameter setting of the algorithm simulation.

N	η (Initial)	η (Final)	Δη
10	28.6	44.6	16%
20	42.3	72.7	30.4%
30	50.3	86.7	36.4%
40	56.7	88.9	32.2%
50	62.7	91.2	28.5%

## Appendix A

- $P_{1}$, $P_{2}$ and $P_{5}$:
  If we assume that the coordinate of the sensor *P* is (px,py,pz), we can determine the XOY coordinates of $P_{1}$, $P_{2}$ and $P_{5}$ on the plane of $pz-PP_{1}$ as follows.
  (A1) $$\begin{array}{c} P_{1}=\left(x_{p_{1}},\;y_{p_{1}}\right)=\left(x_{p},\;y_{p}\right);\\ P_{2}=\left(x_{p_{2}},\;y_{p_{2}}\right)=\left(x_{p}+P_{1}P_{2}\cdot \cos\left(\theta -∠P_{2}P_{1}F_{1}\right),\;y_{p}+P_{1}P_{2}\cdot \sin\left(\theta +∠P_{2}P_{1}F_{1}\right)\right);\\ P_{5}=\left(x_{p_{5}},\;y_{p_{5}}\right)=\left(x_{p}+P_{1}P_{5}\cdot \cos\left(\theta +∠P_{2}P_{1}F_{1}\right),\;y_{p}+P_{1}P_{5}\cdot \sin\left(\theta +∠P_{2}P_{1}F_{1}\right)\right). \end{array}$$
- $\left(x_{r_{a}^{\prime }},\;y_{r_{a}^{\prime }}\right)$, $\left(x_{r_{b}^{\prime }},\;y_{r_{b}^{\prime }}\right)$, $\left(x_{r_{c}^{\prime }},\;y_{r_{c}^{\prime }}\right)$, $\left(x_{r^{\prime }d},\;y_{r^{\prime }d}\right)$:
  As shown in Figure 4c, the turning points of the $R_{i,j}^{\prime }$ on the X–axis are indicated by $\left(x_{r_{a}^{\prime }},\;y_{r_{a}^{\prime }}\right)$, $\left(x_{r_{b}^{\prime }},\;y_{r_{b}^{\prime }}\right)$, $\left(x_{r_{c}^{\prime }},\;y_{r_{c}^{\prime }}\right)$, $\left(x_{r^{\prime }d},\;y_{r^{\prime }d}\right)$ on the plane of $z=z_{p_{1}}$. Utilizing a similar triangular methodology, we can calculate these coordinates, as follows.
  (A2) $$\begin{array}{c} \left(x_{r_{a}^{\prime }},y_{r_{a}^{\prime }}\right)=\left(\left(i-1\right)\cdot R_{w},\;\left(i-1\right)\left(2j-1\right)\cdot P_{2}P_{5}/10\right);\\ \left(x_{r_{b}^{\prime }},y_{r_{b}^{\prime }}\right)=\left(\left(i-1\right)\cdot R_{w},\;\left(i-1\right)\left(2j-3\right)\cdot P_{2}P_{5}/10\right);\\ \left(x_{r_{c}^{\prime }},y_{r_{c}^{\prime }}\right)=\left(i\cdot R_{w},\;i\left(2j-3\right)\cdot P_{2}P_{5}\right);\\ \left(x_{r_{d}^{\prime }},y_{r_{d}^{\prime }}\right)=\left(i\cdot R_{w},\;i\left(2j-1\right)\cdot P_{2}P_{5}\right); \end{array}$$
- $\left(x_{r_{a}},\;y_{r_{a}}\right)$, $\left(x_{r_{b}},\;y_{r_{b}}\right)$, $\left(x_{r_{c}},\;y_{r_{c}}\right)$, $\left(x_{r_{d}},\;y_{r_{d}}\right)$:
  Considering the angle θ of the sensor, all the coordinates of $r_{a},\;r_{b},\;r_{c},\;r_{d}$ of $R_{i,j}$ are calculated based on an anticlockwise rotation $r_{a}^{\prime },\;r_{b}^{\prime },\;r_{c}^{\prime },\;r_{d}^{\prime }$ by θ with respect to the positive direction of the X–axis using the rotation matrix, as follows.
  (A3) $$\left(\begin{array}{c} x_{r}\\ y_{r} \end{array}\right)=\left(\begin{array}{cc} \cos\theta & -\sin\theta \\ \sin\theta & \cos\theta \end{array}\right)\cdot \left(\begin{array}{c} x_{r^{\prime }}\\ y_{r^{\prime }} \end{array}\right)$$
  where $\left(x_{r},y_{r}\right)$ represents the XOY coordinates of $\left(x_{r_{a}},\;y_{r_{a}}\right)$, $\left(x_{r_{b}},\;y_{r_{b}}\right)$, $\left(x_{r_{c}},\;y_{r_{c}}\right)$, $\left(x_{r_{d}},\;y_{r_{d}}\right)$ and $\left(x_{r^{\prime }},\;x_{r^{\prime }}\right)$ represents the XOY coordinates of $\left(x_{r_{a}^{\prime }},\;y_{r_{a}^{\prime }}\right)$, $\left(x_{r_{b}^{\prime }},\;y_{r_{b}^{\prime }}\right)$, $\left(x_{r_{c}^{\prime }},\;y_{r_{c}^{\prime }}\right)$, $\left(x_{r^{\prime }d},\;y_{r^{\prime }d}\right)$.
- $D_{1}$, $D_{2}$, $P_{3}$ and $P_{4}$:
  The XOY coordinate of $D_{1}$, $D_{2}$, $P_{3}$, and $P_{4}$, can be calculated as follows.
  (A4) $$P_{1}D_{2}=P_{1}D_{1}=P_{1}F_{1}/\cos\left(∠P_{2}P_{1}F_{1}\right)$$
  (A5) $$\begin{array}{c} D_{1}=\left(x_{d_{1}},\;y_{d_{1}}\right)=\left(x_{p_{5}},\;y_{p_{5}}\right)\cdot P_{1}D_{2}/P_{1}P_{2}\\ D_{2}=\left(x_{d_{2}},\;y_{d_{2}}\right)=\left(x_{p_{2}},\;y_{p_{2}}\right)\cdot P_{1}D_{2}/P_{1}P_{2} \end{array}$$
  According to Equation (5), we can calculate that
  (A6) $$\begin{array}{c} P_{3}=\left(x_{p_{3}},\;y_{p_{3}}\right)=\left(x_{d_{2}}+D_{2}P_{3}\cdot \cos\theta ,\;y_{d_{2}}+D_{2}P_{3}\cdot \sin\theta \right)\\ P_{4}=\left(x_{p_{4}},\;y_{p_{4}}\right)=\left(x_{d_{1}}+D_{2}P_{3}\cdot \cos\theta ,\;y_{d_{1}}+D_{2}P_{3}\cdot \sin\theta \right) \end{array}$$
